# Perception of children and mothers regarding dental aesthetics and orthodontic treatment need: a cross-sectional study

**DOI:** 10.1186/s40510-016-0149-6

**Published:** 2016-11-14

**Authors:** Emerson Tavares de Sousa, Beatriz Feitosa da Silva, Fabiana Barros Marinho Maia, Franklin Delano Soares Forte, Fábio Correia Sampaio

**Affiliations:** 1 Department of Pediatric Dentistry, Piracicaba Dental School, Campinas University, 901 Limeira Avenue, Areião, Piracicaba, SP 13414-90 Brazil; 2 Department of Clinic and Social Odontology, School of Dentistry, Federal University of Paraiba (Cidade Universitária, s/n), Joao Pessoa, Paraiba 58051-900 Brazil

**Keywords:** Malocclusion, Child, Parents, Orthodontics

## Abstract

**Background:**

The normative orthodontic treatment need, established by dental professionals during the dental appointment, becomes ineffective when it does not evaluate all the factors that influence the decision-making process, including individuals’ perception and satisfaction with their dental appearance. Therefore, the purpose of this study was to investigate the perception of children and their mothers as regards orthodontic treatment need and satisfaction with dental aesthetics and test if these variables are associated with the objective orthodontic treatment needs, assessed by the Dental Aesthetic Index (DAI).

**Methods:**

A cross-sectional study was conducted on 308 children aged 12 years, and their mothers were randomly selected by cluster sampling (primary schools). The variables “orthodontic treatment need,” “satisfaction with chewing,” and “dental appearance” were assessed by means of a questionnaire. The questions were answered individually at school or home, in cases of children or mothers, respectively. DAI was assessed to make an objective clinical assessment. The variables were dichotomized and statistically analyzed by the chi-square and Fisher’s exact tests, contingency *coefficient C*, and logistic regression.

**Results:**

The results of the clinical evaluation (DAI) were statistically associated with the perception of orthodontic treatment need and satisfaction with dental appearance in children (*p* ≤ 0.01). However, no association was observed with regard to satisfaction with chewing and DAI (*p* = 0.10). The children’s perception of orthodontic treatment need and satisfaction with the appearance of their teeth was statistically associated (*p* ≤ 0.01) with their mothers’ perception. Maxillary overjet, maxillary and mandibular misalignment, and dental crowding were associated with the orthodontic treatment need by children and their mothers, with *p* value −0.05 and 5 % level of significance. Maxillary overjet was a significant predictor for the perception of orthodontic treatment need in children (OR 1.86, 95 % CI 0.98–3.55) and mothers (OR 3.02, 95 % CI 1.54–5.92).

**Conclusions:**

Children and parents realize the need for orthodontic treatment according to the different types of malocclusion, as noted in the association between orthodontic treatment need and dental appearance perceived by children and their mothers, which was also observed—with low correlation—with regard to DAI.

## Background

Malocclusion has become a highly prevalent public health problem that influences the quality of life in different ways [1]. Thus, understanding of the health-disease process requires a more contextualized approach that involves issues related to access to health care and decision-making of all those participating in the treatment [2–4].

Several studies have investigated malocclusion, in particular Badran et al. [2], Moura et al. [4], Farias et al. [5], Feu et al. [6], Peres et al. [7], and Thomaz et al. [8], who found several associations. Gender, age, educational level, social status, malocclusion severity, access to dental care, self-perception of facial aesthetics, dentists’ recommendations, concern of parents, and the influence of classmates are commonly associated factors.

The multifaceted of children and their parents’ connotation of malocclusion makes orthodontic planning difficult; therefore, the formulation of a comprehension model should analyze socioeconomic, cultural, and psychosocial factors [2, 3] that might affect the self-perception and aesthetic appearance, as well as the desire for orthodontic treatment need. Therefore, studying the predictors of perceptions is important to enable the planning of health services and oral health promotion, particularly in countries where oral health care is provided by public funds. In addition, the mere clinical view of the disease limits the need for treatment and aesthetic expectations.

The relationship between parents/children and the orthodontic treatment need has been investigated [9]; however, the literature is scarce as regards studies in which the children’s perceptions are compared with those of their mothers. Thus, this study was conducted with the aim of considering the epidemiological variation of malocclusion and the need to understand the relations between those seeking treatments.

The purpose of this study was to investigate if the perception of children and their mothers as regards orthodontic treatment needs and their satisfaction with dental aesthetics and test if these variables are associated with objective orthodontic treatment need according to the Dental Aesthetic Index (DAI) [10].

## Methods

The present study adheres to the STROBE statement (Strengthening the Reporting of Cross-Sectional Studies) [11].

### Sample characteristics

A cross-sectional study was carried out involving a random selection of 12-year-old children of both genders, enrolled in primary public schools in the Brazilian Northeastern region. Sample randomization was obtained by cluster sampling in two stages, in which schools were the primary sampling unit and children were randomly selected from each school as a second stage. The estimated sample size was 308 pupils, assuming the population proportion of 0.50 of orthodontic treatment need, alpha of 0.05 for two-sided confidence level, Estimated Design Effect (DEFF) of 1, and desire level of absolute precision of 0.05 with 95 % significance.

### Eligibility criteria

The sample was randomly selected by drawing of codes assigned to each pupil, based on the list of schoolchildren enrolled and in the age group of interest. If the selected pupil could not be reached, the examiners would return to school another three times. All 12-year-old schoolchildren could participate in the study; however, the exclusion criteria were history of orthodontic treatment, children whose mothers were illiterate, mothers that did not sign the consent form, and children who refused to be examined.

### A pilot study

The examiner was trained and calibrated in a population with same age of the sample. The training exercise consisted of two steps (theoretical and clinical):

- The first step involves a theoretical discussion of the DAI criteria for malocclusion diagnosis. This procedure involved the analysis of photographs, orthodontic dental models, and WHO guidelines.
- The second step was performed to assess the consistency of the clinical analysis. An experienced orthodontist (gold standard) trained one dentistry student using a sample of 20 children, randomly selected. The examiner’s reliability was checked twice within a 2-week period and assessed by Pearson correlation coefficient test. The reproducibility of diagnosis was determined by intra-rater reliability (*r* = 0.84; *p* < 0.01).

A pilot study was carried out with 30 children and mothers to test the methodology and comprehension of the questionnaire. Children and mothers in the pilot study were not included in the main study. The results of the pilot study not revealed misunderstandings regarding the questionnaire or any need to make changes in the collection process.

### Clinical and non-clinical data collection

Previously contacted to attend a school meeting, parents were informed regarding the aim of the research. The mothers who agreed to participate in the study signed a consent form and were instructed to answer the questionnaire.

The questionnaire was structured with categorized questions (yes or no) and variables of interest, including questions about self-perception, appearance satisfaction, and orthodontic treatment need. The sequence of questions for children was (1) identification (name, gender, and mother’s name), (2) Do you feel the need for orthodontic treatment? (3) Are you satisfied with your chewing? and (4) Are you satisfied with the appearance of your teeth? The mothers were asked the following questions: (1) identification (name, age, and child’s name), (2) Do you feel that your child is in need of orthodontic treatment? and (3) Are you satisfied with the appearance of your child’s teeth? The questions were answered individually at school or home, in cases of children or mothers, respectively.

The children were examined at school, according to the WHO recommendations for epidemiological oral health surveys [12]. The child seated in front of the examiner, used individual protection equipment, a sterilized mouth mirror, a WHO probe (Golgran Ind. E Com. Ltda., São Paulo, SP, Brazil), and a gauze to dry the teeth. DAI was assessed to classify malocclusion and the orthodontic treatment need.

The clinical parameters of DAI were based on the measurement of 10 occlusal traits (diastema, maxillary and mandibular overjet, crowding, spacing, maxillary and mandibular misalignment, molar relation, anterior open bite, and crossbite) multiplied by a weight [11, 12]. The severity of malocclusion and weight of DAI determines the orthodontic treatment need by dichotomized scores including no need for treatment (score of ≤25) and defined malocclusion with need for treatment (scores of >25) [13].

### Statistical analysis

Statistical analysis was performed using SPSS software 21.0 (SPSS, Inc., Chicago, IL, USA). The chi-square (*χ* ^2^) and Fisher’s exact tests were performed to test the association between variables, as well as the odds ratio (OR). The contingency coefficient *C* was calculated to quantify the relationship between two nominal variables in the contingency table. The significance level was set at *p* < 0.05 (two-tailed). The multivariate logistic regression assessed the relative influence of types of malocclusion on the perception of orthodontic treatment need using dichotomized variables. The OR and *p* value were calculated to perform a predictive model. Considering the biological plausibility, two models of explanation were executed:

- Model I included all covariables that can be associated with the primary outcome.
- Model II was performed based on statistical significance (*p* < 0.20) of model I and represented the adjusted covariables, with predictive value significant when *p* ≤ 0.05.

## Results

The response rate was 99 % (*n* = 306) and 94 % (*n* = 290) to children and mothers, respectively. These losses were due to the absence of the child at school on oral examination day, refusal to be examined, and when mothers did not returned the questionnaire. The sample was composed of 191 (62 %) boys and 117 (38 %) girls. The prevalence of malocclusion and orthodontic treatment need (DAI > 25) was 76.3 %, with girls showing statistically significant higher prevalence (43.4 %) (*p* = 0.00). Gender was associated with satisfaction with dental appearance in children and their mothers, as well as the objective need for orthodontic treatment—DAI (*p* < 0.05). However, this association was not observed with regard to the desire for orthodontic treatment.

Table 1 shows associations between different types of malocclusion and the perception of children and their mothers as regards treatment need. The need for orthodontic treatment in children and their mothers was associated with maxillary overjet, dental crowding, and maxillary and mandibular misalignment (*p* ≤ 0.05).

**Table 1:** Association between type of malocclusion and perceived orthodontic treatment need in 12-year-old children and their mothers

Type of malocclusion (DAI)	Treatment need (children’s perception)						Treatment need of their child (mothers’ perception)					
	No		Yes		Odds ratio IC (95 %)	*p*	No		Yes		Odds ratio IC (95 %)	*p*
	n	%	n	%			*n*	%	*n*	%		
Diastema												
No	67	21.9	172	56.2	0.56	0.09	67	23.1	158	54.5	0.12	0.12
Yes	12	3.9	55	18	(0.28–1.11)		13	4.5	52	17.9	(0.30–1.15)	
Maxillary overjet												
≤3 mm	57	21.2	119	44.2	0.43	0.00^a^	61	24	103	40.6	0.31	0.00^a^
>3 mm	16	5.9	77	28.6	(0.23–0.81)		14	5.5	76	29.9	(0.16–0.59)	
Mandibular overjet												
No	78	25.5	223	72.9	0.71	1.00 EF	80	27.6	204	70.7	-	0.32 EF
Yes	1	0.3	4	1.3	(0.07–6.49)		0	0	6	1.7		
Crowding												
No	46	15	69	22.5	0.313	0.000^a^	43	14.8	63	21.7	0.369	0.00^a^
Yes	33	10.8	158	51.6	(0.185–0.532)		37	12.8	147	50.7	(0.217–0.626)	
Spacing												
No	55	18	147	48	0.802	0.432	53	18.3	138	47.6	0.976	0.93
Yes	24	7.8	80	26.1	(0.462–1.392)		27	9.3	72	24.8	(0.567–1.682)	
Maxillary misalignment												
No	54	17.6	103	33.7	0.385	0.000^a^	55	19	93	32.1	0.361	0.00^a^
Yes	25	8.2	124	40.5	(0.224–0.661)		25	8.6	117	40.3	(0.209–623)	
Mandibular misalignment												
No	53	17.3	115	37.6	0.504	0.011^a^	51	17.6	105	36.2	0.569	0.03^a^
Yes	26	8.5	112	36.6	(0.295–0.861)		29	10	105	36.2	(0.335–0.966)	
Molar relation												
No	38	12.4	103	37.7	0.896	0.675	34	11.7	100	34.5	1.230	0.43
Yes	41	13.4	124	40.5	(0.537–1.497)		46	15.9	110	37.9	(0.732–2.068)	
Anterior open bite												
No	76	24.8	204	66.7	0.350	0.102 EF	78	26.9	187	64.5	0.208	0.02EF^a^
Yes	3	1	23	7.5	(0.102–1.200)		2	0.7	23	7.9	(0.048–0.906)	
Crossbite												
No	63	20.6	167	54.6	0.707	0.274	63	21.7	156	53.8	0.78	0.42
Yes	16	5.2	60	19.6	(0.379–1.318)		17	5.9	54	18.6	(0.420–1.447)	

*EF* exact Fisher’s test

^a^Significant chi-square test

The multivariate logistic regression analysis is presented in Table 2. The adjusted model shows that maxillary overjet (OR 1.86, 95 % CI 0.98–3.55), spacing (OR 1.92, 95 % CI 1:04–3:56), and crowding (OR 3.46, 95 % CI 1.94–6.18) might be considered as significant predictors for the occurrence of perceived of orthodontic treatment need in children (*p* < 0.05). Regarding mothers’ perception, model II represents that the maxillary overjet (OR 3.02, 95 % CI 1.54–5.92) and maxillary misalignment (OR 0.42, 95 % CI 0.23–0.74) were significant predictor variables (*p* < 0.05); however, the factor that contributed more to mother’s complaint was the anterior open bite, with an increase of six times (OR 6.02, IC 95 % 1.35–26.78).

**Table 2:** Logistic regression of perception of orthodontic treatment need in children and their mothers and the types of malocclusion

Type of malocclusion	Treatment need/children				Treatment need of their child/mothers			
	Model I		Model II		Model I		Model II	
	Odds ratio IC (95 %)	*p* valor	Odds ratio adjusted IC (95 %)	*p* valor	Odds ratio IC (95 %)	*p* valor	Odds ratio adjusted IC (95 %)	*p* valor
Diastema								
No	1.30 (0.46–3.67)	0.61	–	–	1.89 (0.66–5.41)	0.23	–	–
Yes								
Maxillary overjet								
≤3 mm	0.75 (0.07–7.83)	0.05	1.86 (0.98–3.55)	0.05	0.33 (0.16–0.65)	0.00	3.02 (1.54–5.92)	0.00
>3 mm								
Mandibular overjet								
No	0.51 (0.26–1.00)	0.81	–	–	0.00	0.99	–	–
Yes								
Crowding								
No	3.09 (0.93–10.2)	0.06	3.46 (1.92–6.18)	0.00	1.57 (0.45–5.44)	0.47	–	–
Yes								
Spacing								
No	1.77 (0.86–3.62)	0.11	1.92 (1.04–3.56)	0.03	1.14 (0.56–2.34)	0.71	–	–
Yes								
Maxillary misalignment								
No	1.10 (0.43–2.84)	0.83	–	–	1.82 (0.71–4.64)	0.20	0.42 (0.23–0.74)	0.00
Yes								
Mandibular misalignment								
No	0.93 (0.38–2.26)	0.87	–	–	1.03 (0.39–2.67)	0.94	–	–
Yes								
Molar relation								
No	0.93 (0.53–1.62)	0.81	–	–	0.62 (0.35–1.11)	0.11	1.53 (0.87–2.68)	0.13
Yes								
Anterior open bite								
No	2.97 (0.83–10.6)	0.09	3.05 (0.86–10.88)	0.08	5.90 (1.31–26.5)	0.02	6.02 (1.35–26.7)	0.01
Yes								
Crossbite								
No	1.19 (0.59–2.39)	0.61	–	–	0.99 (0.48–2.00)	0.97	–	–
Yes								

Overall statistics were significant with *p* < 0.00

Model II of treatment need/children was adjusted to the variables maxillary overjet, crowding, spacing and anterior open bite

Model II of treatment need of their child/mothers was adjusted to the variables maxillary overjet, maxillary misalignment, molar relation, and anterior open bite

Table 3 shows a significant association (*p* < 0.01) between the objective examination, assessed by DAI, and the dental appearance from the perspective of children and their mothers and between DAI and the perception of children and mothers about orthodontic treatment need. There was no association between DAI and chewing satisfaction.

**Table 3:** Distribution of treatment need (DAI) and association with the satisfaction of chewing, appearance and perception of the child's treatment needs and their mothers

		Treatment need (DAI)				p	CC
		No		Yes			
Children	Satisfaction with Chewing	n	%	n	%		
	Yes	50	16.2	136	44.2	0.10	-
	No	23	7.5	99	32.1		
	Satisfaction with Appearance						
	Yes	40	13.0	70	22.7	0.00^a^	0,21^b^
	No	33	10.7	165	53.6		
	Treatment Need						
	Yes	38	12.4	189	61.8	0.00^a^	0,26^b^
	No	34	11.1	45	14.7		
Mothers	Satisfaction with their child’s Appearance						
	Yes	31	10.3	53	17.5	0.00^a^	0,19^b^
	No	40	13.2	178	58.9		
	Treatment Need of their child						
	Yes	32	11.0	178	61.4	0.00^a^	0,27^b^
	No	34	11.7	46	15.9		

*DAI* Dental Aesthetic Index, *CC* Contingency Coefficient C

^a^ Chi-square test significant ^b^ Correlation is significant at the 0.00 level (1-tailed)

Table 4 shows the association between the perception of children and their mothers as regards orthodontic treatment need (*p* = 0.000), and a pattern of agreement between yes (65 %) and no (18 %) was found. Similarly, self-satisfaction with the appearance of teeth and the mothers’ satisfaction of a child’s appearance were significantly associated (*p* < 0.01).

**Table 4:** Association of perceived of treatment need and satisfaction with appearance between children and their mothers

Treatment Need/ Children	Treatment Need of their child/ Mothers					
	Yes		No		*p*	CC
	*n*	%	*n*	%		
Yes	188	65.3	29	10.9	0.00*	0,48**
No	20	6.9	51	17.7		
Satisfaction with Appearance/ Children	Satisfaction with their child’s Appearance/ Mothers					
	Yes		No		*p*	CC
	*n*	%	*n*	%		
Yes	56	18.5	52	17.2	0.00*	0,37**
No	28	9.3	166	55		

*CC* Contingency Coefficient C

* Chi-square test significant ** Correlation is significant at the 0.00 level (1-tailed)

## Discussion

This study used clinician exams to assess the DAI [10] to simulate the dental analysis. This index attributes a mathematical score that reflects the severity of malocclusion and uses a cut-off point to determine the need for orthodontic treatment focusing on the aesthetic aspects of occlusion [14]. There are possible limitations with the use of the DAI as well as the other orthodontic treatment need indices; however, these indices can be used in epidemiological research and in treatment planning [15].

The perception of orthodontic treatment need was not associated with gender, in contrast to the treatment need according to DAI, which provides information depending on the variable (OR = 2.44, 95 % CI = 1.33–4.45, *p* = 0.03). Furthermore, gender can be associated with dental appearance according to the mothers and children’s perception, so it was noted that mothers of girls tended to be dissatisfied with the dental appearance of their daughters, which could be observed with regard to the child’s self-perception. This issue is controversial in the literature, and there are studies that either prove this association [16, 17] or not [18, 19]. However, the variability in the methodological approach of these studies is well known, with different designs, samples, and indicators.

The types of malocclusion were associated with certain components of the DAI in both groups of individuals. Dental crowding, maxillary overjet, and maxillary and mandibular misalignment were directly associated with the need perceived in 12-year-old children and their mothers (Table 1). Dias and Gleiser [16] observed this fact in a similar sample, but with a different data collection methodology. There is an agreement between the perception of orthodontic treatment need and some types of malocclusion, with propensity for the most prevalent anterior disorders. Moreover, changes in occlusion might cause dissatisfaction with appearance and influence psychological health in terms of self-concept, quality of life, and treatment decision [4, 20, 21]. When the types of malocclusion were analyzed by a logistic regression, the maxillary overjet, spacing, and crowding were predictable variables for orthodontic treatment need in children, with chances of 1.86, 1.93, and 3.46, respectively. On the other hand, the maxillary overjet and anterior open bite increased at three and six times the chance to the negative perception of mothers regarding the child’s necessity of treatment. The adjustment of multivariate logistic regression minimized the number of covariables performing a plausible and clinically generalized model, without confounding factors (overall statistics *p* < 0.00).

Satisfaction with the appearance of teeth and the perception of orthodontic treatment need showed the same trend in both groups studied, with significant association. This study found that children and their mothers more frequently realize the need for orthodontic treatment, which might be associated with satisfaction with the appearance of teeth and suggested a possible impact of these children’s oral conditions on their mothers’ lives. Badran et al. [2] found the association between self-perception of dental aesthetics and the perception of mothers about their children’s dental appearance and orthodontic treatment need.

The literature is contradictory with regard to parental knowledge about their children, especially on issues related to activities outside the home and deepest feelings [4]. Jokovic et al. [22] observed that many parents reported not knowing about issues related to their children’s welfare. However, orthodontic treatment need might be directly connected to aesthetics, determined by cultural and social issues, suggesting that there should be consensus between parents’ and children’s perception of what is considered satisfactory.

The ideal orthodontic treatment might be overestimated by clinicians when compared to patients’ smile perception [23]. There is an association between what it is evaluated clinically and what it is bothering the child, but these aspects must be evaluated with caution. There is a low correlation between what it is determined by the dentist (DAI) and the treatment need perceived by children and mothers. Comparably, Spalj et al. [24] showed a positive significant association between objective (DAI) and subjective assessment of orthodontic treatment need in children, in addition to showing that the aesthetic evaluation of parents had low predictive value.

The high prevalence of dissatisfaction with appearance and the subjective need for orthodontic treatment was relevant in this study. This fact might be attributed to body changes, increasing interest in dating and relationships, which makes body and physical attributes especially important at this stage of development [4, 7]. Shalish et al. [25] reported that self-satisfaction with dental appearance had a greater effect on the self-perception of orthodontic treatment need in pediatric patients.

Additionally, it is important to emphasize that socioeconomic issues might also influence individuals’ perceptions. This study comprised a population with low socioeconomic level and high need for orthodontic intervention (76.3 %) in 12-year-old children. Piovesan et al. [26] showed that parents with low incomes were more likely to categorize the oral health of their children as “poor,” but they did not explain the relationship of this dissatisfaction with malocclusion. This is important to emphasize that social disparities might be associated with this perception. Such inequalities could affect the well-being of families and children, resulting in a negative impact due to psychosocial and environmental influences and even to material deprivation [27].

The association between the need for orthodontic treatment (DAI) and the child’s satisfaction with chewing was not significant, suggesting that malocclusion has no direct impact on the perception of masticatory function, as was as also observed by Tessarollo et al. [20]. Therefore, the limitation of the method should be emphasized, because it failed to assess changes that caused greater masticatory discomfort such as overbite, posterior tooth loss, open bite, and posterior cross bite [5].

This study did not evaluate the social impact of malocclusion and all the variables that influence the need for orthodontic treatment in children; in this case, it focused only on factual and not causal relations. More importantly, the present findings, based on a representative sample, can be used to formulate hypotheses for populations with similar demographic and cultural characteristics, low socioeconomic status, and living in an underdeveloped country.

It is important to recognize the limitations of the present study. First, the inherent limitations of cross-sectional studies in which exposure and outcome are determined simultaneously, and the time sequence is often impossible to define. Secondly, the risk of bias from the answers on the questionnaires is also important to consider. Therefore, to minimize this latter limitation, a pilot study was performed.

Clinically, it is important to emphasize that the subjective nature of DAI, which depends on the cultural aspects of the country, was limited for assessing the psychosocial aspects of oral health and the quality of life of the individuals [28]. Thus, the use of a personal questionnaire that includes the perception of children and parents regarding the orthodontic treatment need is necessary before formulating a treatment plan, for a more contextualized and effective approach.

## Conclusions

Children and mothers realize the desire for undergoing orthodontic treatment according to some types of malocclusion; similarly, they perceived the orthodontic treatment need. Furthermore, associations were observed between the subjective treatment need and dental appearance perceived by children and their mothers, which also might be found with regard to DAI, but with low correlation.
